# Parsonage-Turner Syndrome Post-COVID-19 Oxford/AstraZeneca Vaccine Inoculation: A Case Report and Brief Literature Review

**DOI:** 10.7759/cureus.34710

**Published:** 2023-02-06

**Authors:** Sofia Meixedo, Miguel Correia, Ana Machado Lima, Ismael Carneiro

**Affiliations:** 1 Physical Medicine and Rehabilitation, Centro Hospitalar Vila Nova de Gaia/Espinho - Centro de Reabilitação do Norte, Gaia, PRT

**Keywords:** covid-19 vaccine complication, covid-19, brachial neuritis, neurologic amyotrophy (na), parsonage-turner syndrome (pts)

## Abstract

Parsonage-Turner syndrome (PTS) is a rare brachial plexus neuropathy that typically presents as a severe, sudden-onset pain followed by atrophic weakness with slow recovery, which may occur after an identifiable triggering event. Vaccination is one of several known triggers of PTS, and this syndrome has already been reported in other patients who were vaccinated against coronavirus disease.

We report the case of a 75-year-old Caucasian man who received the third dose of the coronavirus disease 2019 (COVID-19) Oxford/AstraZeneca vaccine and was diagnosed with PTS. A week after inoculation, the patient, with no history of trauma, developed a sudden-onset left shoulder mechanical pain and later reported an abduction deficit. Neurological examination showed an atrophy of the proximal muscles of the left upper limb. No bulbar weakness or pathological upper motor neuron signs were seen. The MRI excluded rotator cuff pathology, including ruptures and tendinopathy. Electroneuromyography findings carried out 10 months after the onset of symptoms indicated left brachial panplexopathy, suggestive of PTS.

The raised consciousness of PTS and vaccine association is crucial for prompt identification and diagnosis and, therefore, better clinical outcomes.

## Introduction

Parsonage-Turner syndrome (PTS), otherwise known as paralytic brachial neuritis, idiopathic brachial plexopathy, idiopathic neuralgic amyotrophy, brachial plexus neuropathy, and acute brachial radiculitis, was first described in 1948 by Parsonage et al. after observing 136 patients during neurological work in the Army in the United Kingdom and in the India Command [1]. It is a rare peripheral neuropathy that affects the brachial plexus, characterized by severe sudden-onset pain followed by atrophic weakness with slow recovery [2]. Even though PTS typically presents with severe pain, painless forms have been described with an identical disease course [2].

Symptoms may be preceded by a triggering event, such as an infection, surgery, strenuous exercise, or, less commonly, vaccination. However, nearly half of the affected individuals have no identifiable triggering event [3]. The upper trunk of the brachial plexus with the suprascapular nerve and the long thoracic, axillary, and anterior interosseous nerves are the most commonly affected [3]. Involvement of the phrenic nerve was also reported, with the most frequent symptoms being dyspnea with effort, sleep disorders, and orthopnea. That said, to implement proper treatment, it is very important to be alert to these symptoms in any patient diagnosed with idiopathic brachial plexopathy; non-invasive mechanical ventilation may be needed [4].

Although PTS has been reported after COVID-19 infection and vaccination, there are currently few published cases. We describe a case report of PTS that developed days after the administration of the COVID-19 Oxford/AstraZeneca vaccine (ChAdOx1-S [recombinant] vaccine).

## Case presentation

Initial presentation

A 75-year-old Caucasian man with hypertension, dyslipidemia, and benign prostatic hyperplasia, and with no history of trauma, developed a sudden-onset mechanical left shoulder pain, quantified 7 out of 10 on a numeric pain rating scale, a week after he was inoculated with the COVID-19 Oxford/AstraZeneca vaccine in his left arm. Nevertheless, he experienced only mild symptoms attributed to the vaccine. He then developed a shoulder abduction and flexion deficit. Faced with these complaints, the patient went to his general practitioner (GP) and was prescribed a 10-day course of non-steroidal anti-inflammatory drugs (NSAIDs), which did not improve his condition. As the symptomatology remained unchanged, the GP referred him to physical medicine and rehabilitation (PMR) consultation, but only six months after the onset of symptoms.

Six months after the onset of symptoms

**Figure 1:**
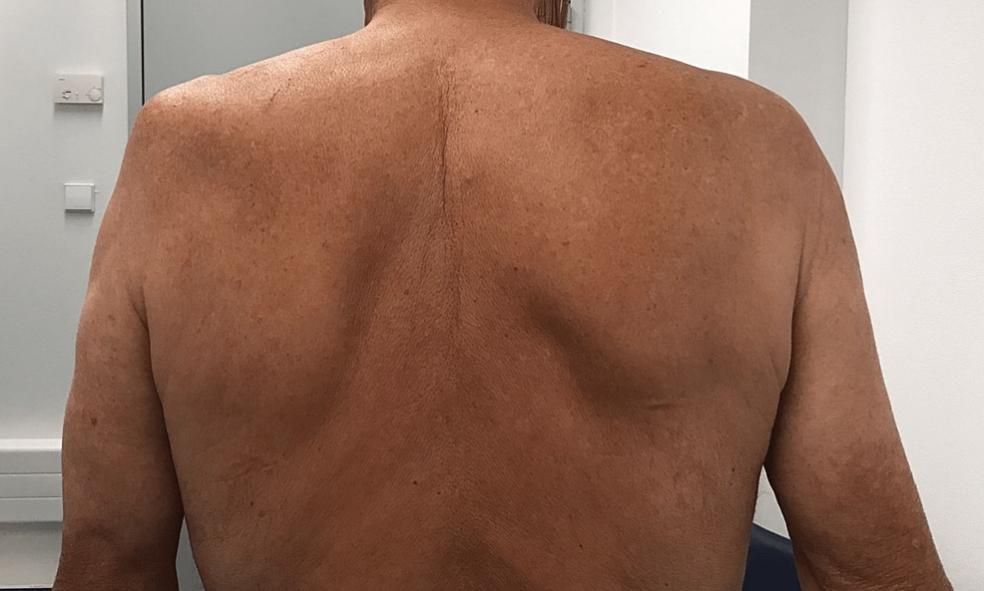
Six months after the onset of symptoms before the physiatric rehabilitation program; supraspinatus muscle atrophy and lateral scapular winging

**Figure 2:**
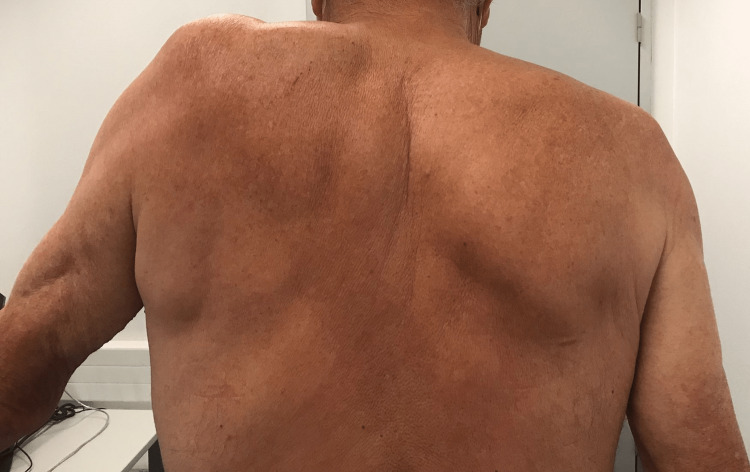
Six months after the onset of symptoms before the physiatric rehabilitation program; shoulder abduction of approximately 20 degrees

At that time, the patient quantified his mechanical left shoulder pain as 3 out of 10 on a numeric pain rating scale, with the pain considerably residual compared with the initial presentation. He denied numbness or sensitivity impairment of the limb. At physical examination, it was observed a supraspinatus muscle atrophy and lateral scapular winging (Figure 1). Active range of motion was severely limited, as the patient couldn’t flex or abduct the shoulder more than 20 degrees (Figure 2). Passive shoulder range of motion was intact.

Regarding specific rotator cuff tests, the subscapularis muscle appeared intact, with the internal rotation lag sign and lift-off test being negative. During supraspinatus muscle assessment, the Jobe test was slightly painful, and a clear strength asymmetry in comparison with the contralateral side was noticed. Regarding the infraspinatus muscle, the external rotation lag sign was positive, and the patient had pain and strength asymmetry in resisted external rotation. About the teres minor muscle, the patient had a negative Hornblower’s sign but a painful Patte test.

The cervical spine range of motion was preserved and painless. Deep tendon reflexes were present bilaterally and symmetrically. No bulbar weakness or pathological upper motor neuron signs were found. The Spurling test was negative. The shoulder ultrasound performed two months after the onset of symptoms showed a partial insertional tear of the supraspinatus and exuberant left subacromial bursitis.

Thus, the clinical findings were interpreted as a probable rupture of the rotator cuff tear, and the patient was referred to an orthopedic consultation. However, the MRI later requested showed only foci of microtears on the articular surface of the supraspinatus tendon, an increased signal of the infraspinatus tendon caused by probable fibrillar partial rupture of some of the fibers of the internal slope of the middle third of the humeral head. A normal subscapularis tendon and normal trophism of the muscle bellies of the rotator cuff tendons were observed. No other significant anomalies were identified. That said, the patient had no surgical indication and was advised to start classic physiatric treatment and to be reevaluated by PMR.

Eight months after the onset of symptoms

The patient maintained residual pain complaints but markedly reduced joint ranges. At physical examination, a lateral scapular winging and a supraspinatus, infraspinatus, and teres minor atrophy were observed. Other muscles, including the deltoid and periscapular muscles, had full strength. The specialized tests and provocative maneuvers to evaluate the rotator’s cuff and biceps brachialis integrity were similar to the last evaluation.

There were no changes in superficial sensitivity except for the C8 dermatome. Evaluating by specific peripheral nerve, a tactile sensitive alteration was observed in the territory of the radial (C5-T1) and cubital (C8-T1) nerves, but no change was observed in the motor function of these nerves. With these findings arose the possibility that we were facing a PTS, which was why an electromyographic study was requested.

Ten months after the onset of symptoms

Electroneurography and electromyography findings suggested left brachial panplexopathy with more important involvement of the upper trunk of moderate severity, suggestive of PTS.

The patient started a physiatric rehabilitation program (one-hour sessions, twice a week) for 2.5 months. During every session, the patient was subjected to moist heat on periscapular muscles followed by manual therapy to reduce muscle contractures. Meanwhile, transcutaneous electrical nerve stimulation (TENS) at 100 Hz, 200 us per pulse, and paresthesic intensity were used for analgesia. Then, the shoulder joint range of motion was addressed, first by passive joint mobilization, and later by assisted active joint mobilization. Rotator cuff muscle strength was first addressed in isometry (shoulder adducted, elbow flexed at 90 degrees), progressing to concentric contractions with a progressive load. Patient education, focusing on scapulohumeral kinetics and dynamic scapular stabilizers muscles strengthening (serratus anterior, rhomboideus, and trapezius), was also performed. Sensitivity reeducation in the C8-T1 dermatome, dorsal, and volar surface of the hand was pursued. Neuromuscular electric stimulation was used in the deltoid, supraspinatus, and infraspinatus muscles for a faster atrophy recovery, with these muscles considered partially innervated and the currents prescribed according to this rationale (triangular waves, 50 pulses per minute, 175 us per pulse, with 2 seconds of ramp-up and ramp-down, 8 seconds ON-time, and 48 seconds OFF-time).

To evaluate the impact of physical therapy on this patient, the Shoulder Pain and Disability Index (SPADI) scale was applied before starting the treatment sessions. As such, the patient had 44.5% shoulder dysfunction, scoring 22/50 in the pain score and 36/80 in the disability score, having more difficulty in putting on an undershirt or jumper and being incapable of placing an object on a high shelf. Regarding personal hygiene, the patient reported having 5/10 difficulty washing his hair and back.

Twelve months after the onset of symptoms

Two months after the last assessment, the patient was reevaluated in consultation. At this time, supraspinatus, infraspinatus, and teres minor atrophy was still observed. The patient was now able to actively elevate the shoulder to 45 degrees with an important functional impact on hygiene and other day-to-day activities. Paresthesia of the fourth and fifth fingers of the left hand was present.

Fourteen months after the onset of symptoms

**Figure 3:**
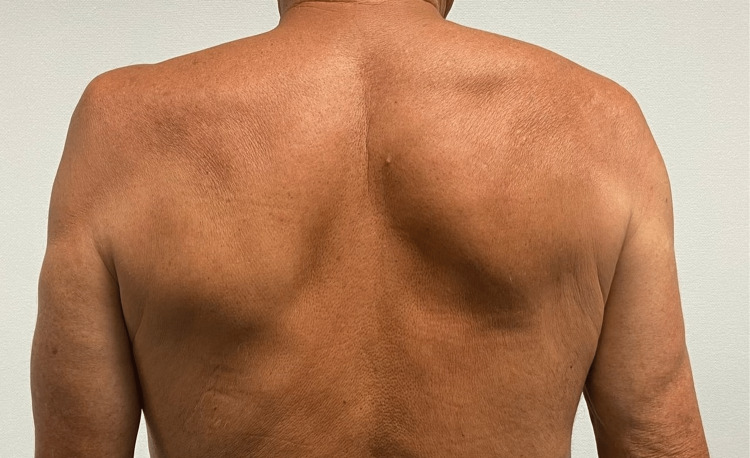
Fourteen months after the onset of symptoms after 2.5 months of the physiatric rehabilitation program; supraspinatus muscle atrophy and lateral scapular winging

**Figure 4:**
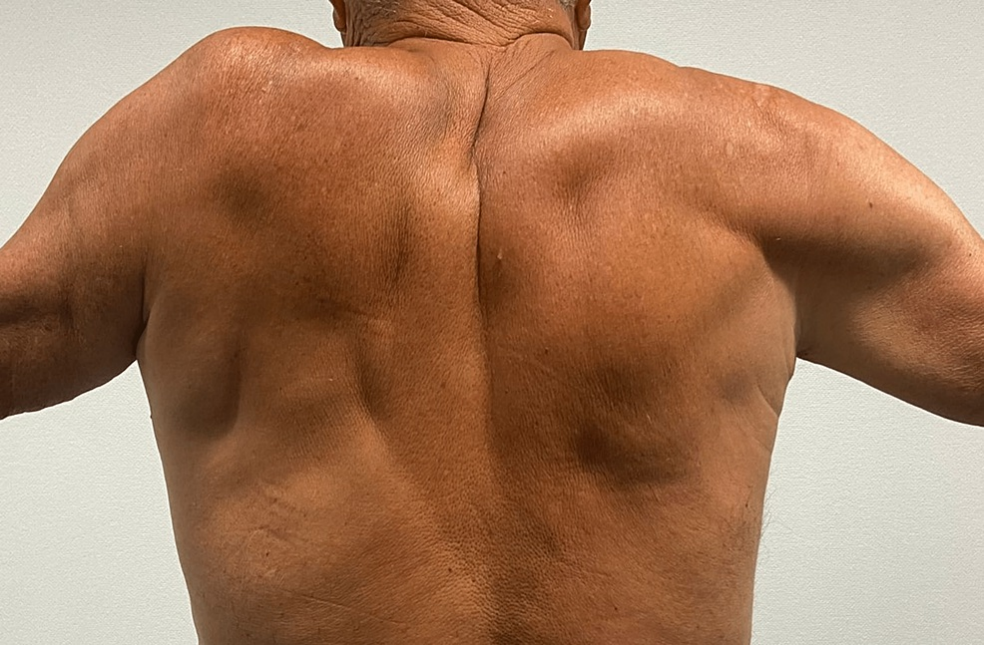
Fourteen months after the onset of symptoms after 2.5 months of the physiatric rehabilitation program; shoulder abduction up to 50 degrees

Once the patient completed the physical therapy program, the muscular atrophy (Figure 3) was still observed, but the patient was now able to actively elevate the shoulder up to 50 degrees, both anteriorly and laterally (Figure 4). At this time, according to the SPADI scale, the patient scored 33% shoulder dysfunction, scoring 14/50 in the pain score and 29/80 in the disability score, as he was still not capable of placing an object on a high shelf and had difficulties on putting on an undershirt or jumper and on removing something from the back pocket. Fourteen months after onset, his range of motion and strength subjectively improved but did not return to baseline levels.

After this, as a clear benefit of physiatric treatment was observed, the patient continued with a new cycle of physiotherapy.

## Discussion

PTS is a rare brachial plexus neuropathy characterized by a sudden-onset, severe pain followed by atrophic weakness with a slow recovery usually preceded by a triggering event such as an infection, surgery, strenuous exercise, or vaccination [3]. We suspected the diagnosis of PTS secondary to COVID-19 vaccine inoculation for the close temporal relationship to vaccine administration. Although we cannot be sure of the causality relationship, vaccination, although rare, is a well-characterized trigger [5], and our patient had no other triggers. Latency time also corroborates our diagnosis hypothesis, as the majority of cases usually occurred one to seven days after the antecedent event [5].

PTS diagnosis confirmation may be challenging, and an alternative and more frequent cause of upper extremity pain can confound the diagnostic algorithm. Subacromial bursitis, adhesive capsulitis, cervical radiculopathy, entrapment neuropathies, facioscapulohumeral dystrophy, and motor neuron disease, are commonly the main differential diagnosis [2].

PTS is primarily a clinical diagnosis supported by electrodiagnostic testing. Nerve conduction studies are supportive and can exclude more common mononeuropathies while needle electromyography is important to document denervation. Imaging may be indicated to exclude the possibility of a mass lesion, especially in those with atypical clinical symptoms or a history suggestive of malignancy [6].

The shoulder ultrasound performed two months after the onset of symptoms in our patient’s case showed a partial insertional tear of the supraspinatus and exuberant left subacromial bursitis, but he never presented pain with inflammatory characteristics or ipsilateral decubitus or nocturnal pain. The rotator cuff's primary function is to hold the humeral head in the glenoid. If the muscles are injured or become weakened, as in our presented case, there is a superior migration of the humeral head within the glenoid, and the subacromial bursa undergoes friction between the humeral head and acromion superiorly.

Findings from diagnostic tests are influenced by tests’ timing. It is not until after two to four weeks of symptoms that MRI or electrodiagnostics can identify the denervation of affected muscles. However, initial diagnostic tests are frequently performed within this period of time to rule out anatomical abnormalities that mimic PTS such as rotator cuff tears and cervical etiologies [6].

PTS’s etiology is still not fully understood, but it is thought to be an immune-mediated reaction against the brachial plexus that occurs in genetically predisposed individuals [7]. Vaccines may trigger a pro-inflammatory response, and an immune-mediated reaction may occur by molecular mimicry and bystander activation most likely facilitated by the disruption of the blood-nerve barrier caused by compression and stretching of the plexus [7]. A direct nerve insult from inoculation leading to PTS is unlikely because PTS after vaccination and other peripheral neuropathies are often reported contralateral to the injected side [8].

Although there are currently no approved treatments, PTS is managed conservatively. Optimization of upper extremity motion and strength is the main treatment goal. Physical therapy may be helpful to preserve the range of motion but will not speed recovery. Additionally, neuromuscular electrostimulation should be considered especially when the denervated state is prolonged, as this therapy plays an important role in the preservation and recovery of muscle mass and function, as it directly stimulates skeletal muscle protein synthesis and primarily recruits fast-twitch (type II) muscle fibers, those with the most significant role in the decline in motor performance and muscle strength. However, early oral prednisone may reduce PTS symptom duration and severity [6]; but in our case reported, given the delay in diagnosis, corticosteroid therapy was not performed in the acute stage.

During the mass COVID-19 vaccination, reporting of more post-inoculation peripheral neuropathies was expected, but the evidence is usually insufficient to link the vaccines directly to these occurrences [8]. According to the literature to date, 15 cases of patients, mostly male and aged between 35 and 66 years, with the onset time of the first symptoms ranging from 18 hours to 35 days after the inoculation, were reported. In almost all patients, there were compatible pathological findings in the electrophysiological study. Also, the cases occurred after the administration of three different COVID-19 vaccines, suggesting that the syndrome can occur despite the vaccine’s mechanism of action [9-20].

**Table 1:** Clinical and demographic characteristics of PTS cases associated with the COVID-19 vaccination up to the date of publication published in the literature in English and available for free reading yo: years old; NA: not available

STUDY	COVID-19 VACCINE	SEX	AGE	TIME ONSET OF FIRST SYMPTOMS	PLEXUS INVOLVEMENT	PAIN	MOTOR SYMPTOMS	SENSORY SYMPTOMS	EMG FINDINGS	TREATMENT	CLINICAL IMPROVEMENT
Crespo B.J. et al., 2021 [9]	AstraZeneca	Male	38 yo	4 days	3 trunks	YES	Neurological examination did not reveal any motor or sensory deficit		YES	Corticosteroids	YES
Carlo C. et al., 2022 [10]	Pfizer	Female	50 yo	2 days	Isolated musculocutaneous nerve involvement	YES	Weakness in the right biceps brachii	Sensation diminished to light touch throughout the right C5 dermatome	YES	Corticosteroid + Pregabalin + Occupational therapy	NA
Koh et al., 2021 [13]	Pfizer	Male	50 yo	25 days	Upper and middle trunk (ipsilateral to the injection site, right)	YES	Weakness and numbness of the arm and lateral forearm		NO	Corticosteroids	YES
	Pfizer	Male	44 yo	4 days	Lower trunk	YES	Hand weakness	Right medial forearm and hand numbness	YES	No corticosteroids	YES
	Moderna	Male	58 yo	35 days	Lower trunk	YES	Distal hand weakness and numbness		YES	Corticosteroids	YES
Diaz-et al., 2022 [14]	Pfizer	Female	35 yo	9 days	Upper trunk	NO	Left arm weakness, numbness, and paresthesias		YES	Corticosteroids	YES
Mahajan et al., 2021 [15]	Pfizer	Male	50 yo	1 week	NA	YES	Left-hand grip and left-wrist extension weakness	NO	YES	Corticosteroids + Occupational therapy	YES
Bernheimer et al., 2022 [16]	Moderna	Female	42 yo	3 weeks	Upper trunk	YES	NO	Paresthesia radiating down the left upper extremity	YES	Corticosteroids + Gabapentin + Physical therapy	YES
Vitturi et al., 2021 [20]	AstraZeneca	Male	51 yo	4 days	NA	YES	Muscle weakness on abduction and elevation of the left upper limb	Hypoesthesia	YES	NSAID + Pregabalin + Physiotherapy	YES
Queler et al., 2022 [8]	Pfizer	Male	49 yo	13 hours	NA	YES	Mild weakness in forearm pronation and wrist flexion	Numbness in the middle volar forearm region	YES	Corticosteroids	YES
	Moderna	Male	44 yo	18 days	NA	YES	Inability to abduct the left shoulder beyond 20 degrees and weakness in external rotation	Hyperesthesias in the left lateral shoulder and diminished sensation to pinprick in the radial nerve distribution	YES	Gabapentin + Physical therapy	YES

## Conclusions

PTS should be considered a diagnostic hypothesis in a patient with severe shoulder pain and weakness after the administration of a COVID-19 vaccine and may be especially relevant as worldwide COVID-19 vaccination is now a reality. Its etiology is thought to be an immune-mediated reaction against the brachial plexus occurring in genetically predisposed individuals, but the cases published up to this date describe PTS after the administration of at least three different COVID-19 vaccines, suggesting that the syndrome can occur despite the vaccine’s mechanism of action.

PTS diagnosis confirmation may be challenging, and common shoulder pathology can mimic PTS and confound the diagnostic algorithm. Findings from diagnostic tests are influenced by their timing, as not until after two to four weeks MRI or electrodiagnostics can identify nerve involvement. PTS is managed with physical therapy, but early oral prednisone may reduce its symptom duration and severity. That said, it is important to enhance that early diagnosis, and adequate therapy may help shorten the course of the disease.

## References

[1] Parsonage M, Turner J (1948). Neuralgic amyotrophy the shoulder-girdle syndrome. Lancet.

[2] van Alfen N (2011). Clinical and pathophysiological concepts of neuralgic amyotrophy. Nat Rev Neurol.

[3] Van Eijk JJ, Groothuis JT, Van Alfen N (2016). Neuralgic amyotrophy: an update on diagnosis, pathophysiology, and treatment. Muscle Nerve.

[4] van Alfen N, Doorduin J, van Rosmalen MH (2018). Phrenic neuropathy and diaphragm dysfunction in neuralgic amyotrophy. Neurology.

[5] van Alfen N, van Engelen BG (2006). The clinical spectrum of neuralgic amyotrophy in 246 cases. Brain.

[6] Bromberg MB (2022). Brachial plexus syndromes. UpToDate.

[7] Gstoettner C, Mayer JA, Rassam S (2020). Neuralgic amyotrophy: a paradigm shift in diagnosis and treatment. J Neurol Neurosurg Psychiatry.

[8] Queler SC, Towbin AJ, Milani C, Whang J, Sneag DB (2021). Parsonage-Turner syndrome following COVID-19 vaccination: MR neurography. Radiology.

[9] Crespo Burillo JA, Loriente Martínez C, García Arguedas C, Mora Pueyo FJ (2021). Amyotrophic neuralgia secondary to Vaxzevri (AstraZeneca) COVID-19 vaccine. Neurologia (Engl Ed).

[10] Civardi C, Delconte C, Pisano F, Collini A, Geda C (2022). Isolated musculocutaneous involvement in neuralgic amyotrophy associated with SARS-CoV2 vaccination. Neurol Sci.

[11] Lukács K, Csőregh É, Fekete B (2022). Bilateral Parsonage-Turner syndrome after COVID-19 vaccination. A case report and review of the literature [Article in Hungarian]. Orv Hetil.

[12] Kim SI, Seok HY, Yi J, Cho JH (2021). Leg paralysis after AstraZeneca COVID-19 vaccination diagnosed as neuralgic amyotrophy of the lumbosacral plexus: a case report. J Int Med Res.

[13] Koh JS, Goh Y, Tan BY (2021). Neuralgic amyotrophy following COVID-19 mRNA vaccination. QJM.

[14] Diaz-Segarra N, Edmond A, Gilbert C, Mckay O, Kloepping C, Yonclas P (2022). Painless idiopathic neuralgic amyotrophy after COVID-19 vaccination: a case report. PM R.

[15] Mahajan S, Zhang F, Mahajan A, Zimnowodzki S (2021). Parsonage Turner syndrome after COVID‐19 vaccination. Muscle Nerve.

[16] Bernheimer JH, Gasbarro G (2022). Parsonage Turner syndrome following vaccination with mRNA-1273 SARS-CoV-2 vaccine. J Clin Neuromuscul Dis.

[17] Flikkema K, Brossy K (2021). Parsonage-Turner syndrome after COVID-19 vaccination: a case report. JBJS Case Connect.

[18] Coffman JR, Randolph AC, Somerson JS (2021). Parsonage-Turner syndrome after SARS-CoV-2 BNT162b2 vaccine: a case report. JBJS Case Connect.

[19] Mitry MA, Collins LK, Kazam JJ, Kaicker S, Kovanlikaya A (2021). Parsonage-turner syndrome associated with SARS-CoV2 (COVID-19) infection. Clin Imaging.

[20] Vitturi BK, Grandis M, Beltramini S, Orsi A, Schenone A, Icardi G, Durando P (2021). Parsonage-Turner syndrome following coronavirus disease 2019 immunization with ChAdOx1-S vaccine: a case report and review of the literature. J Med Case Rep.

